# High-Content Screening of a Taiwanese Indigenous Plant Extract Library Identifies *Syzygium simile* leaf Extract as an Inhibitor of Fatty Acid Uptake

**DOI:** 10.3390/ijms19072130

**Published:** 2018-07-22

**Authors:** Chia-Hung Yen, Hsun-Shuo Chang, Tsai-Hsun Yang, Sheng-Fan Wang, Ho-Cheng Wu, Yu-Chang Chen, Kai-Jay Lin, Sheena Wang

**Affiliations:** 1 Graduate Institute of Natural Products, College of Pharmacy, Kaohsiung Medical University, Kaohsiung 80708, Taiwan; hschang@cc.kmu.edu.tw (H.-S.C.); bakerc313@gmail.com (T.-H.Y.); u104831005@kmu.edu.tw (H.-C.W.); poping73@gmail.com (K.-J.L.); sirius00422@gmail.com (S.W.); 2 Center for Infectious Disease and Cancer Research, Kaohsiung Medical University, Kaohsiung 80708, Taiwan; wasf1234@kmu.edu.tw; 3 Research Center for Natural Products & Drug Development, Kaohsiung Medical University, Kaohsiung 80708, Taiwan; 4 Department of Medical Research, Kaohsiung Medical University Hospital, Kaohsiung 80708, Taiwan; 5 School of Pharmacy, College of Pharmacy, Kaohsiung Medical University, Kaohsiung 80708, Taiwan; yuchang@kmu.edu.tw

**Keywords:** non-alcoholic fatty liver disease, lipid droplet, high-content screening, metabolic diseases, natural products

## Abstract

Non-alcoholic fatty liver disease (NAFLD) has become the most common liver disease in the recent decades in both developed and developing countries, and is predicted to be the major etiology for liver transplantation in the next decade. Thus, pharmacological strategies to treat NAFLD are urgently needed. Natural products are considered an excellent source for drug discovery. By utilizing an image-based high-throughput screening with a library containing 3000 Taiwanese indigenous plant extracts, we discovered that the extract of *Syzygium simile* leaves (SSLE) has an anti-lipid droplet (LD) accumulation effect in hepatic cell lines. Analyses of the expression profile of genes involved in lipid metabolism revealed that SSLE suppressed the mRNA expression of *CD36*, fatty acid translocase. In agreement with this observation, we showed that SSLE inhibited CD36 protein expression and fatty acid uptake and has only limited effects on pre-formed LDs. Moreover, SSLE reduced LD accumulation and *CD36* expression in enterocyte and macrophage cell lines. In conclusion, our findings suggest that SSLE could serve as a potential source for the discovery of novel therapeutic modalities for NAFLD and that the suppression of *CD36* expression and fatty acid uptake could contribute to the lipid-lowering effect of SSLE.

## 1. Introduction

Non-alcoholic-fatty liver disease (NAFLD) is a spectrum of liver diseases that includes: simple steatosis, with fatty accumulation in >5% of hepatocytes; non-alcoholic steatohepatitis (NASH)‒steatosis with inflammation; hepatic fibrosis; and, ultimately, cirrhosis, in individuals who consume little (less than 20 g/day for women and 30 g/day for men) or no alcohol [1]. NAFLD is the most common cause of chronic liver disease in developed and developing countries. The prevalence of NAFLD is ~15% in Europe, ~33% in the USA [2,3,4], and 12–55% in Taiwan [5,6]. The prevalence of NAFLD has increased dramatically worldwide due to the adoption of sedentary lifestyles and globalization of the western diet [7,8]. NAFLD may become the major etiology for liver transplantation by the 2020s [7]. Evidence is accumulating that NAFLD is not only strongly associated with metabolic disorders—including type 2 diabetes mellitus (T2DM), cardiovascular disease (CVD), chronic kidney disease (CKD), and hepatocellular carcinoma (HCC)—but also increased risks and overall mortality of these diseases [9]. The escalating prevalence of NAFLD has increased the demand for medical therapy. The primary treatment for NAFLD is elimination of the underlying risk factors. Reducing body weight by ~5% significantly improves the liver condition, however, subsequent weight gain frequently occurs leading to the recurrence of NASH [10]. No approved drugs are currently available for NAFLD treatment, thus NAFLD remains a major unmet clinical need [11]. Therefore, novel pharmacological strategies against NAFLD are urgently needed.

The term hepatic steatosis (fatty liver), the very first stage of NAFLD, refers to an intracellular accumulation of lipids in the cytoplasm of hepatocytes. The excess lipids in the liver are stored as natural lipids such as triglyceride (TG) and cholesterol esters in dynamic organelles named lipid droplets (LDs). LDs serve as a reservoir of membrane lipid and provide a buffer during times of energy deprivation [12]. An imbalance between formation and degradation of LDs leads to severe physiological consequences including lipodystrophy, NAFLD, obesity, CVD, and T2DM [13]. Therefore, LD homeostasis could be a critical target for the treatment of abnormal lipid accumulation and for the progression of liver diseases in NAFLD patients [14]. 

As natural products are evolutionarily optimized for interactions with biomolecules, they are an invaluable source of drug discovery [15,16,17]. Here, a collection of 3000 Taiwanese indigenous plant (TIP) extracts was used as a natural product library for a high content screening (HCS) for anti-NAFLD drug discovery. The extract prepared from the leaves of *Syzygium simile* (SSLE) was able to reduce the accumulation of LDs in several cell types including liver, intestine, and macrophage. We also uncovered a potential mechanism of action of SSLE.

## 2. Results

### 2.1. High-Throughput Image-Based Screening Identifies Syzygium simile leaf Extract (SSLE) as a Suppressor of Lipid Accumulation

**Figure 1:**
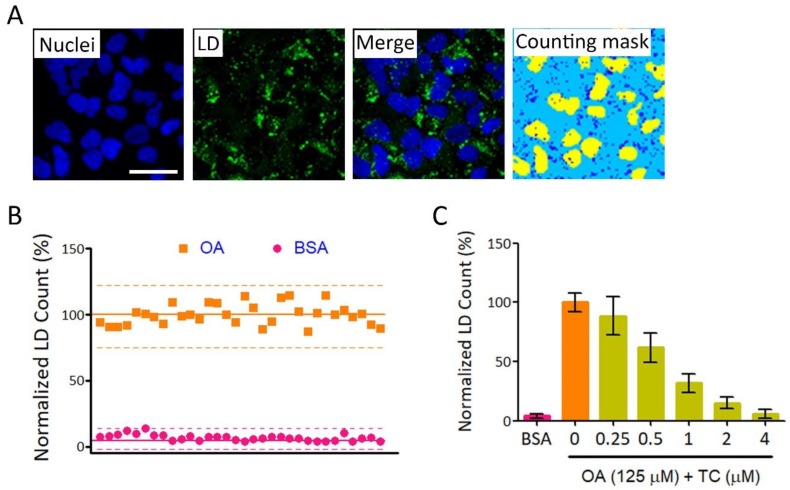
Establishment of a high-throughput screening (HTS) platform for anti-fatty liver drug screening. (**A**) Huh7 cells were used as a cell model for lipid accumulation by treating with 125 μM oleic acid (OA) for 24 h. LD numbers were determined as described in Section 4. Nuclei and lipid droplets (LDs) were stained with Hoechst 33342 (blue) and BODIPY^®^ 493/503 (green), respectively. A granularity analyzing module was used to identify nuclei and LDs automatically. Yellow and blue masks the identified nuclei and LDs, respectively. Bar: 50 μm; (**B**) Variation test for platform evaluation. LDs/cell were determined in three experimental groups: OA (squares) and bovine serum albumin (BSA) (circles). In each group, 32 wells were tested. The average of LD counts in the OA group was used as a standard for 100%. Each dot represents a mean relative LD count from nine fields for one well. Solid lines represent the mean relative LD count and dashed lines represent ± 3 standard deviation (SD) for each group; (**C**) The platform was tested using Triacsin C (TC) as an inhibitor of LD accumulation. LD assay was performed as described in (**A**) in the absence or presence of TC at indicated concentration.

We used an LD assay and a HCS instrument to establish a high-throughput screening (HTS) platform for anti-NAFLD drug discovery. Figure 1A shows typical LD formation and a counting mask generated by the HCS instrument. The average LD counts/cell of bovine serum albumin (BSA)-conjugated oleic acid (OA) + drug vehicle (DMSO)-treated wells (hereinafter referred to as OA) were used as standard for 100% of fatty loading. The quality of this platform was evaluated by S/N (signal-to-noise ratio), S/B (signal-to-background ratio), and Z’-factor. For S/N and S/B calculation, fatty acid free BSA + drug vehicle (DMSO)-treated wells (hereinafter referred to as BSA) were considered the background, while OA-treated wells were defined as the signal. For Z’-factor calculation, OA-treated wells were defined as general sample group, because the majority of compounds in a library have very low or no biological activity, thus, the average measurement would be close to that of vehicle control. As an inhibition type assay, BSA-treated wells that showed minimum signals were defined as negative control [18]. The S/N, S/B, and Z’-factor for this platform were 37.8, 14.6, and 0.66, respectively (Figure 1B and Table 1). These results suggested that our platform is good quality and can be used for HTS. Triacsin C (TC) is a potent inhibitor of acyl-CoA synthetase long-chain family member 1 (ACSL1). ACSL1 catalyzes the formation of fatty acyl-CoA, which can be used to synthesize TG and phospholipids. TC has been reported to inhibit fatty acid-induced LD formation in cultured human hepatocytes [19]. We found that TC reduced LDs accumulation dose-dependently in our platform (Figure 1C).

**Figure 2:**
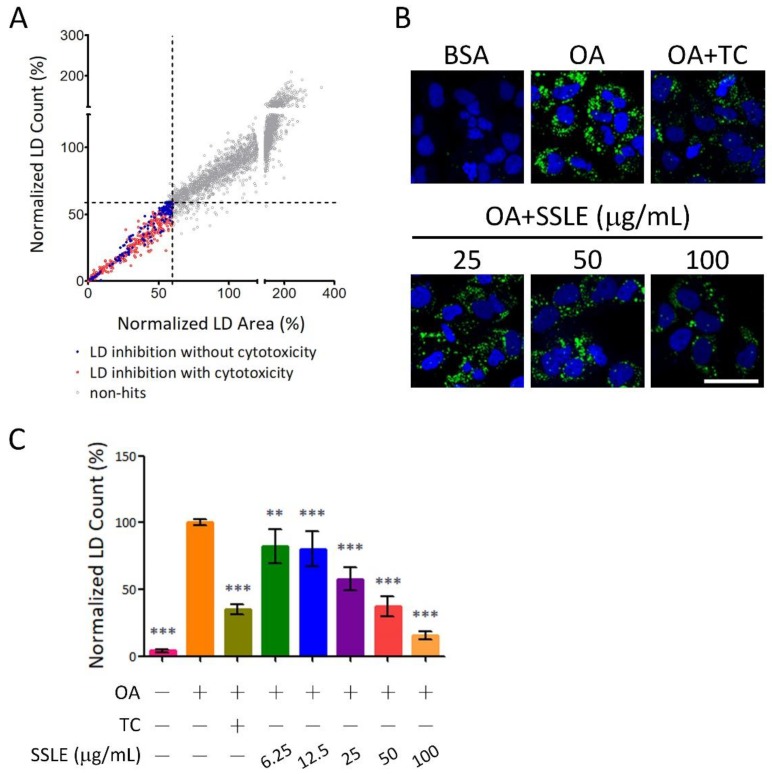
Identification of *Syzygium simile* leaf extract (SSLE) as an inhibitor of lipid accumulation. The platform described in Figure 1 was used for HTS. The screening flowchart is shown in Figure S2. (**A**) The XY-scatter plot shows the distribution of normalized LD count and area in primary screening. Each dot represents the mean of four fields from one well treated with one extract from the Taiwanese indigenous plant (TIP) library. Extracts that reduced LD formation by >40% (LD content <60%, which was approximately equivalent to <4.5 SDs (SD of all OA control wells); dashed lines) are indicated by red dots. Extracts that fitted the criteria without severe cytotoxicity (cell count >60%) were primary screen hits (blue dots). The results of re-validation are shown in Figure S3; (**B**,**C**) Huh7 cells were treated with BSA or OA with the indicated concentrations of SSLE or 1 μM TC for 24 h. (**B**) Representative images; (**C**) Quantification results. Bar: 50 μm. * significant difference from OA control group (** *p* < 0.01, *** *p* < 0.001, compared with controls using the one-way analysis of variance (ANOVA) and Dunnett’s tests).

The screening flowchart is described in Figure S2. As number and size are important parameters for LD homeostasis, both LD number and area were measured and applied as screening criteria. Extracts that reduced both LD count and area >40% without severe cytotoxicity (cell count >60% average cell count of control wells) were hits in the primary screen (Figure 2A). A total of 141 extracts were selected for secondary screening, and 20 for further validation in three concentrations. Extracts that reduced LD content in a dose-dependent manner and >50% at 50 μg/mL were final hits for HTS. After validation, we identified *Syzygium simile* leaf extract, *Syzygium formosanum* leaf extract, and *Sterculia nobilis* leaf extract as hits of HTS; *Syzygium simile* leaf extract (SSLE) was the most potent candidate with >40% LD content reduction at 25 μg/mL (Figure S3 and Figure 2B,C). 

### 2.2. SSLE Exerts Anti-LD (Lipid Droplet) Formation Activity in Other Liver Cell Lines

**Figure 3:**
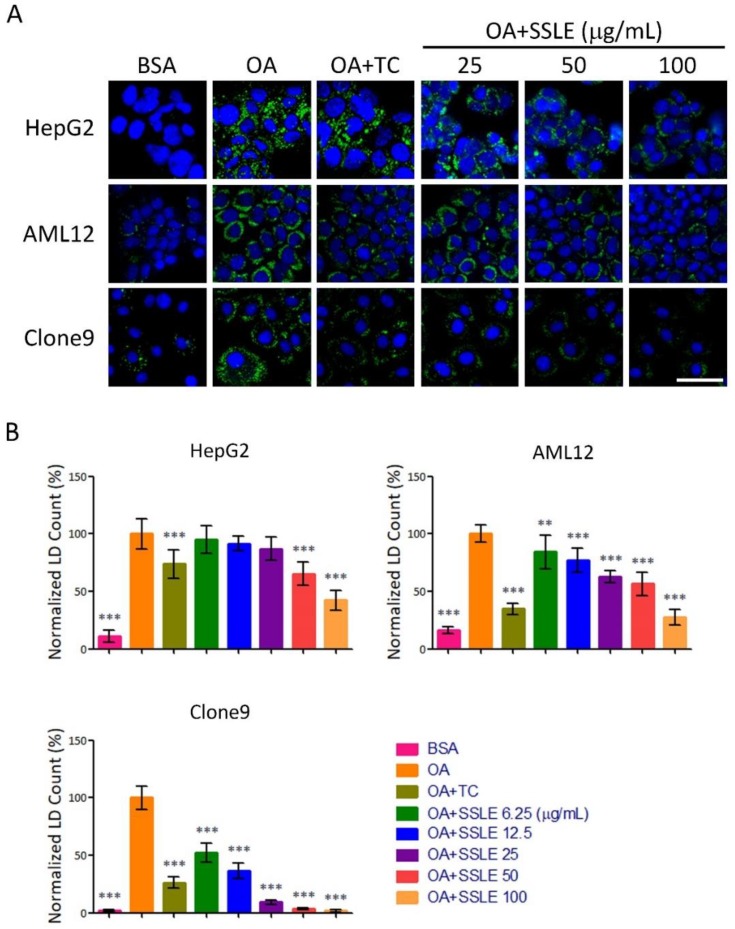
SSLE exerted anti-LD formation activity in other liver cell lines. Three hepatic-lineage cell lines from HepG2 (human), AML12 (mouse), and Clone9 (rat) cells were used to examine the anti-lipid accumulating effect of SSLE by LD assay. Cells were treated as described in Figure 2. Representative images are shown in (**A**) and quantification results in (**B**). Bar: 50 μm. * significant difference from OA control group (** *p* < 0.01, *** *p* < 0.001, compared with controls using the one-way ANOVA and Dunnett’s tests).

We tested the effects of the extract on LD accumulation in other liver cell lines from human (HepG2), mouse (AML12), and rat (Clone9). SSLE reduced LD accumulation dose-dependently in these three cell lines and in Huh7 cells (Figure 3). Next, we determined the half inhibitory concentration for LD inhibition (IC_50_) and for cell cytotoxicity (CC_50_) of SSLE. The IC_50_ for SSLE in these four cell lines ranged from 7.6 to 77 μg/mL. The CC_50_ of SSLE ranged from 81 to >200 μg/mL (Table 2 and Figure S4). We calculated the selective index by determining the ratio of CC_50_ to IC_50_. The selective indexes for Huh7, HepG2, AML12, and Clone9 were 5.5, 2.6, 1.6, and 23 respectively. The results suggest that SSLE causes only mild cytotoxicity with acceptable anti-LD accumulating activity, thus SSLE is a potential lead source for anti-NAFLD drug discovery worth developing further.

### 2.3. SSLE Reduces mRNA and Protein Expression of CD36 

**Figure 4:**
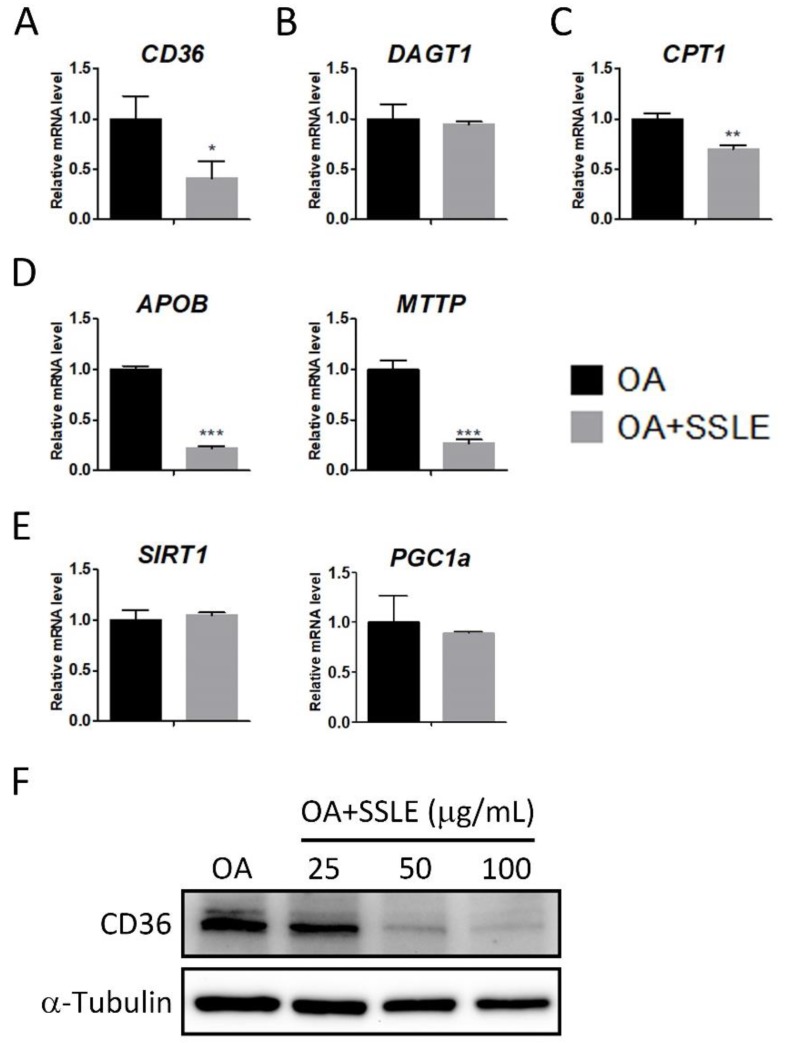
SSLE reduced the mRNA and protein expression of *CD36*. (**A**–**E**) Huh7 cells were treated with 125 µM OA in the presence or absence of 100 µg/mL SSLE for 12 h. RT-qPCR was used to determine the expression profile of lipid metabolism genes. (**A**) Lipid entry: *CD36*; (**B**) TG formation: *DGAT1*; (**C**) β-oxidation: *CPT1*; (**D**) lipid secretion: *APOB* and *MTTP*; (**E**) mitochondria biogenesis: *SIRT1* and *PGC1a*. * significant difference from the DMSO control group (* *p* < 0.05, ** *p* < 0.01, *** *p* < 0.001, *t*-test); (**F**) Huh7 cells were treated as indicated for 12 h. Protein expression of CD36 was determined by immunoblot.

LDs are dynamic organelles and their homeostasis is influenced by the balance between lipid storage and lipid catabolism. More than half of TG stored in hepatocytes in patients with NAFLD are contributed by fatty acids released from peripheral adipose [20]. Thus, the uptake of these excessive free fatty acids by the liver is critical for NAFLD development. However, decreased lipolysis, β-oxidation, or very-low-density lipoprotein (VLDL) secretion also impairs lipid homeostasis by reducing lipid catabolism [14]. Therefore, to investigate the molecular mechanisms by which SSLE suppresses LD accumulation, we performed qPCR to determine the effects of SSLE on the expression profile of lipid metabolism genes including *CD36*, the fatty acid translocase involved in fatty acid uptake; *DGAT1*, a TG synthesis-associated gene; *CPT1*, the rate-limiting enzyme for β-oxidation; *APOB*, a component of very-low-density lipoprotein; *MTTP*, involves in lipoprotein assembly; *SIRT1* and *PGC1a*, involve in energy homeostasis and mitochondria biogenesis. *CD36* and *DGAT1* regulate lipid storage, while *CPT1*, *APOB*, *MTTP*, *SIRT1*, and *PGC1a* regulate lipid catabolism. SSLE treatment resulted in significant suppression of *CD36*, *APOB*, and *MTTP* expression (Figure 4A–E). The dose-dependent suppressive effect of SSLE on *CD36* mRNA and protein expression was further demonstrated by qPCR and immunoblot (Figure S5 and Figure 4F).

### 2.4. SSLE Reduced Fatty Acid Uptake

**Figure 5:**
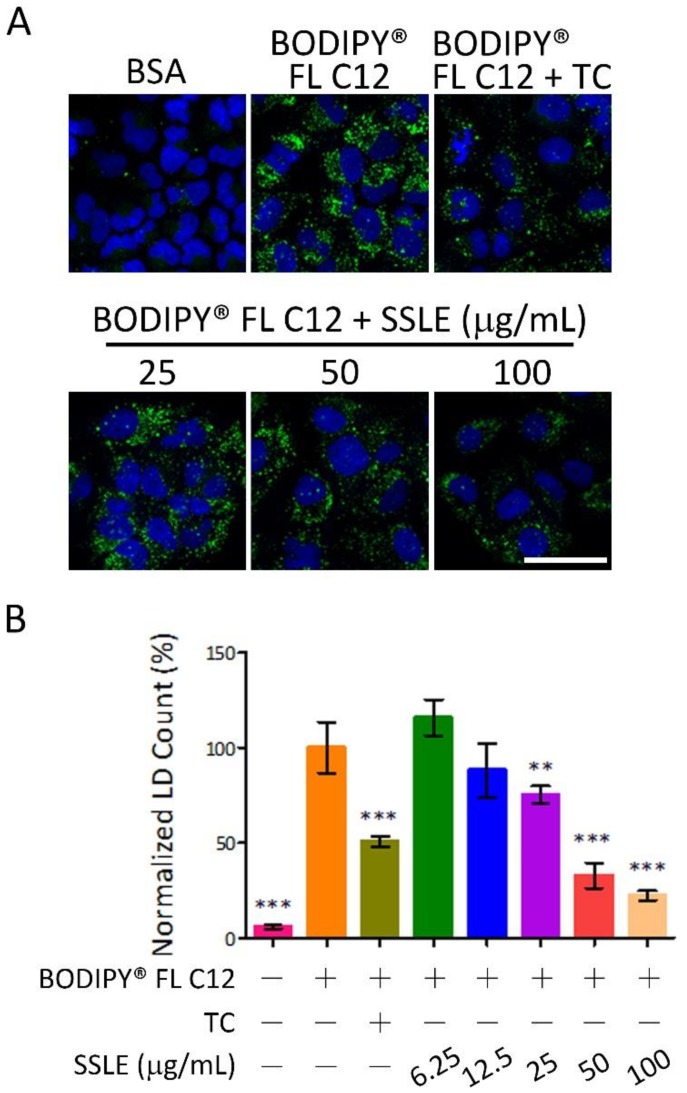
SSLE reduced fatty acid uptake. BODIPY-labeled dodecanoic acid (BODIPY^®^ FL C12) was conjugated to BSA and used as the fatty acid source in the LD assay. Huh7 cells were treated as indicated for 9 h, then fixed and stained with Hoechst 33342. (**A**) Representative images; (**B**) Quantification results. The concentration of BODIPY^®^ FL C12 and TC were 125 µM and 1 µM, respectively. Bar: 50 μm. * significant difference from OA control group (** *p* < 0.01, *** *p* < 0.001, compared with controls using the one-way ANOVA and Dunnett’s tests).

We next addressed whether SSLE prevents LD formation by reducing fatty acid uptake. Fluorescence-labeled dodecanoic acid (BODIPY^®^ FL C12) was used to trace the uptake and incorporation of fatty acid into LDs. BODIPY-labeled dodecanoic acid was conjugated to BSA and used for fatty acid loading in the LD staining assay. Cells were treated for 9 h then harvested for nuclei staining. SSLE reduced fatty acid uptake dose-dependently with an IC_50_ of 28.6 μg/mL (Figure 5).

### 2.5. SSLE Showed Limited Reducing Effects on Pre-Formed LDs

To gain more insight into the molecular mechanisms by which SSLE suppressed LD accumulation, we tested whether SSLE could reduce pre-formed LDs. First, we determined the kinetics of LD formation. Time-lapse images of living cells were acquired once an hour for 24 h by ImageXpress Micro System (Molecular Devices) automatically. The formation of LD reached maximum at 6 h post-OA loading, remained at high level for next 6 h, and reduced progressively 12 h post-OA loading (Figure S6 and Videos S1–S3). 

**Figure 6:**
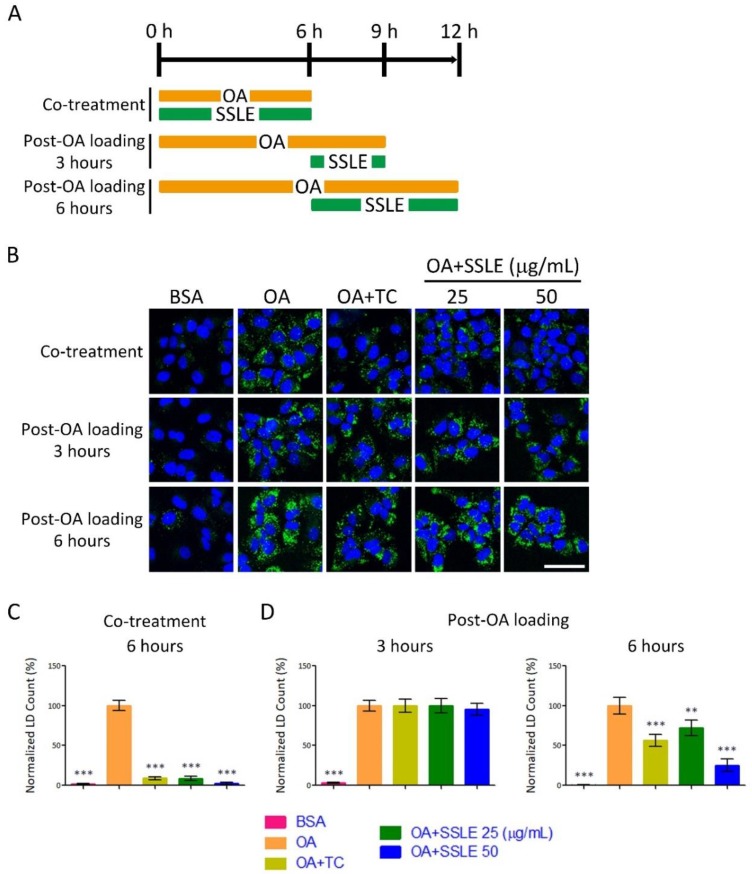
SSLE showed limited reducing effects on pre-formed LDs. (**A**) Scheme of the time-of-adding experiment. Huh7 cells were treated with 125 µM OA and SSLE at the indicated concentrations or 1 µM TC for 6 h (co-treatment protocol) or treated with 125 µM OA for 6 h to allow LD formation, then co-treated with SSLE at the indicated concentrations or 1 µM TC for an additional 3 or 6 h (post-OA loading protocol). (**B**) Representative images. Bar: 50 µm. The quantification results of the co-treatment experiment are shown in (**C**) and of post-OA loading experiments in (**D**). * significant difference from OA control group (** *p* < 0.01, *** *p* < 0.001, compared with controls using the one-way ANOVA and Dunnett’s tests).

Next, we performed a time-of-adding experiment using an LD staining assay to determine the effect of SSLE on pre-formed LDs. The experimental scheme is described in Figure 6A. SSLE, even at the lower concentration of 25 μg/mL, suppressed LD accumulation dramatically when co-treated with OA (Figure 6B,C) within 6 h of treatment. However, SSLE at the same concentration showed no effect on pre-formed LD with 3 h of treatment, and only a limited reduction of pre-formed LDs was observed at this concentration after 6 h of treatment (Figure 6B,D). As an inhibitor of LD formation, TC strongly reduced LD accumulation in co-treatment scheme but not in post-OA loading scheme. Together, these results suggest that SSLE reduces LD accumulation by preventing LD formation. 

### 2.6. SSLE Exerted an LD-Reducing Effect in Other Cell Types

**Figure 7:**
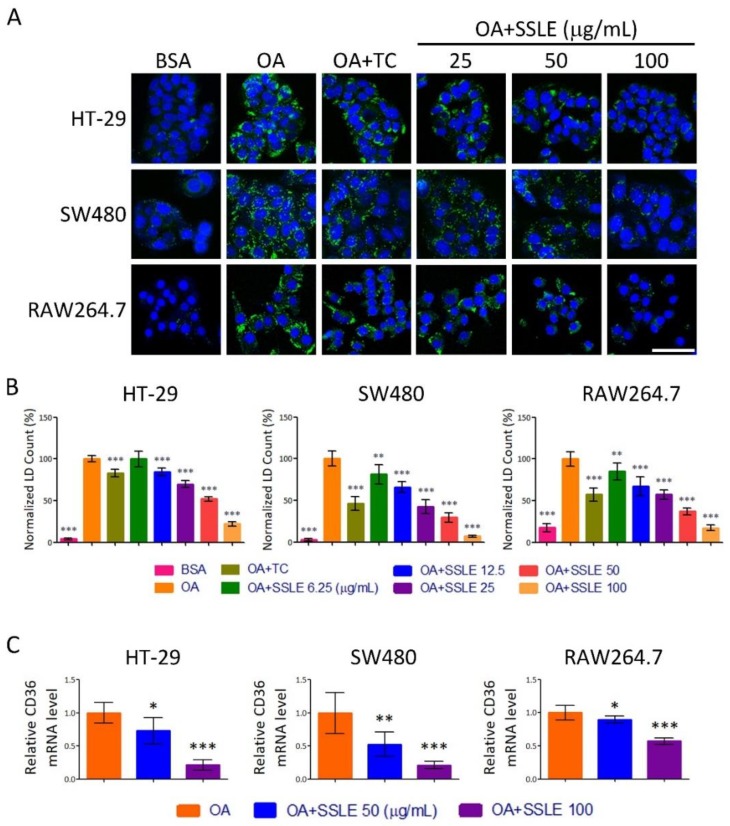
SSLE exerted an LD-reducing effect in other cell types. Three cell lines, including two enterocyte cell lines: HT-29 and SW480 cells and macrophage cell line. RAW264.7 cells were used to examine the anti-lipid accumulating effect of SSLE by LD assay. Cells were treated with BSA or OA (62.5 μM for enterocyte cell lines; 250 μM for RAW264.7 cells) in the absence or presence of the indicated concentrations of SSLE or 1 μM TC. After 24 h, LD counts were determined as described above. (**A**) Representative images. Bar: 50 μm; (**B**) Quantification results; (**C**) The effect of SSLE on *CD36* gene expression in enterocyte and macrophage cell lines. Cells were treated with OA with concentrations described above in the presence or absence of indicated concentration of SSLE for 12 h. A RT-qPCR assay was used for determining the expression of *CD36*. * significant difference from OA control group (* *p* < 0.05, ** *p* < 0.01, *** *p* < 0.001, compared with controls using the one-way ANOVA and Dunnett’s tests).

A western-style diet (high fat and high sucrose) is one of the major factors contributing to the development of NAFLD and metabolic disorders. Enterocytes are the first cells to uptake fatty acid after food intake. Thus, we tested the LD-reducing effect of SSLE on enterocytes. Moreover, in monocyte/macrophages, CD36 plays a critical role in the development of atherosclerotic lesions and is a potential therapeutic target [21,22]. Therefore, we also examined the effect of SSLE in a macrophage cell line. The LD staining assay showed that SSLE reduced LD accumulation dose-dependently in both enterocyte cell lines-HT-29 and SW480 and the macrophage cell line-RAW264.7 (Figure 7A,B). The IC_50_ for HT-29, SW480, and RAW264.7 were 53.6 ± 1.7, 11.8 ± 0.6, and 33.8 ± 4.7 respectively. Furthermore, a significant reduction in the expression of *CD36* in both macrophage and enterocyte cells was observed upon SSLE treatment (Figure 7C). 

## 3. Discussion

The estimated global prevalence of NAFLD is ~30% and is likely to increase [6,9]. As NAFLD is an independent risk factor for other metabolic diseases and no approved drugs are available to treat this disorder, NAFLD is a major unmet clinical need. Currently, two agents targeting hepatic fat accumulation such as obeticholic acid and elafibranor are being evaluated in phase 3 trials for the treatment of NASH [23,24]. Obeticholic acid and elafibranor are agonists for farnesoid X receptor and peroxisome proliferator-activated receptor-α/δ, respectively [23,24]. By utilizing an HTS platform to screen the TIP library, we identified that SSLE possesses anti-NAFLD potential. Our findings suggest a possible mechanism of action of SSLE in which SSLE lowers cellular lipid accumulation by suppressing fatty acid uptake by reducing the expression of *CD36*. 

*Syzygium simile* (Merr.) Merr. (Figure S7) is an evergreen tree distributed in the Philippines and Taiwan [25,26]. *S. simile* belongs to the Myrtaceae family. Myrtaceae is the ninth largest flowering plant family and is found in wet tropics, particularly South America, Australia, and Tropical Asia [27]. Myrtaceae are widely used in human daily life and several species produce edible fruits used to make juice, jelly, and sweets, such as *Psidium guajava* (guava), *Myrciaria cauliflora* (jaboticaba), *Eugenia uniflora* (pitanga), *Syzygium cumini* (jambolan), and *Syzygium samarangense* (wax jambo). Myrtaceae species are also used in folk medicine to treat several diseases, especially gastrointestinal disorders, and hemorrhagic and infectious diseases [25]. Data-mining of Reaxys revealed that 192 compounds had been isolated from Syzygium genus. The majority of these compounds belong to flavonoids, galloyl glucoses, and triterpenoids (Table S2). Anthocyanin/ellagitannin-enriched extracts from *S. cumini* showed high antioxidant and anti-proliferative activities against lung cancer cell line [28]. A polyphenol-rich leaf extract from *S. aqueum* exhibits antioxidant, hepatoprotective, pain-killing, and anti-inflammatory activities in animal models [29]. The ethanol extract of *S. formosanum* showed good free radical scavenging activity and heme oxygenase-1-induced activity [30]. Terpenoids isolated from *S. formosanum* exhibit inhibitory activities against reverse transcriptase of Moloney murine leukemia virus [31]. We showed here for the first time that the methanolic extracts of leaves of *S. simile* and *S. formosanum* possess inhibitory effects on cellular lipid accumulation. To the best of our knowledge, no investigation of the chemical constituents and bioactivity research of *S. simile* has been previously reported. Regarding the clinical needs of anti-NAFLD therapeutic agents, the chemical profile and identification of the active component(s) in *S. simile* are currently under investigation. 

The expression of CD36 is low in normal hepatocytes but is upregulated by lipid-rich diets, hepatic steatosis, and NAFLD [32]. Moreover, CD36 plays an active role in fatty acid uptake and has significant effects on hepatic steatosis and insulin sensitivity [33]. DGAT1 is a rate-limiting enzyme of TG synthesis. Pharmacological inhibition of DGAT1 showed lipid lowering effect and reduced body weight in high-fat diet induced obesity mice without apparent liver damage [34]. CPT1 has been considered the rate-limiting enzyme for β–oxidation. A flavonoid—Baicalin—acts as a natural allosteric activator of CPT1 and ameliorates high-fat diet-induced obesity and hepatic steatosis [35]. MTTP, the APOB chaperone protein, is required for the assembly and secretion of APOB-containing lipoproteins in the liver and intestine. Genetic defect in *MTTP* or *APOB* gene is associated with liver steatosis, obesity, and insulin resistance [36]. SIRT1, a key regulator of energy metabolism, enhances β–oxidation by activating PGC1α. Activation of SIRT1/PGC1α pathway enhances mitochondria biogenesis and hepatic fat catabolism [37]. We showed that SSLE could suppress cellular lipid accumulation by reducing the expression of *CD36*, the fatty acid translocase, and subsequently preventing fatty acid uptake by liver cells. Interestingly, we also found that SSLE reduced lipid accumulation, as well as the expression of *CD36*, in a macrophage cell line and in enterocyte cell lines, which indicates that SSLE may affect the same mechanism in different cell types. CD36 is an important target for treating NAFLD and metabolic syndrome. Our findings underline the therapeutic potency of SSLE or its derivatives for treating NAFLD or other metabolic disorders. In addition, SSLE suppressed the expression of *CPT1*, *MTTP*, and *APOB*, which suggests that lipid oxidation and secretion is unlikely to contribute to the reduction of cellular lipid content. More investigations are needed to clarify whether these phenomena were resulted from the direct effect of SSLE on gene expression or from feedback regulations of reduced lipid intake. Nonetheless, for future studies on SSLE, the issue should be concerned is that the suppressive effect of SSLE on MTTP and APOB could potentially compromise its anti-NAFLD activity. 

Natural products and their derivatives have long been used as medications [38]. Compared to synthetic and combinatorial compounds, natural products are recognized for their high chemical structure diversity, biochemical specificity, and their biologically relevant molecular scaffolds, optimized during evolution [16,17]. Therefore, natural products remain the best source of lead structures for drug discovery. Analyses of new medicines approved by the US Food and Drug Administration revealed that over one third were natural products, direct derivatives of natural products, or were developed based on natural products [15,16,17]. Taiwan is a subtropical island with hills and mountains covering two thirds of its surface. With a changeable climate, Taiwan has tremendous biodiversity. The TIP library established here consists of 3000 extracts from 1336 species of Taiwanese indigenous plants. We believe that this library, which has high chemical diversity, is a useful resource for new drug development.

## 4. Materials and Methods

### 4.1. Construction of the Taiwanese Indigenous Plant Extract Library (TIP Library)

The collection of 3000 methanolic extracts was provided by Professor Ih-Sheng Chen from the Department of Pharmacy, School of Pharmacy, Kaohsiung Medical University. This collection included crude extracts prepared from the root, stem, leaves, flower, or whole plant of 1336 plants originating from Taiwan. The classification characteristics of this library are shown in Table S1. The flowchart of library construction is shown in Figure S1. *S. simile* leaf extract (SSLE) was prepared as following: 10.8 kg of fresh leaves of *S. simile* were collected and air dried to yield 5.48 kg of dried leaves. They were extracted with cold methanol at room temperature three times, with each extraction lasting for three days. The methanol solution was condensed under reduced pressure to give 650 g of methanolic extract.

### 4.2. Cell Lines

Cells were cultured as described previously [39]. The medium for Huh7 and HepG2 is DMEM supplemented with 10% FBS, P/S (penicillin (100 U/mL) and streptomycin (100 μg/mL)), nonessential amino acids (0.1 mM), and l-glutamine (2 mM) (Thermo Fisher Scientific, Waltham, MA, USA). The medium for AML12 is a 1:1 mixture of DMEM and Ham’s F12 medium with 10% FBS and 1× ITS-A supplement (Thermo Fisher Scientific). The medium for Clone9 and RAW264.7 is DMEM supplemented with 10% FBS and P/S. The medium for HT29 is McCoy’s 5a medium (modified) supplemented with 10% FBS and P/S. SW480 was cultured in Leibovitz’s L-15 medium supplemented with 10% FBS and P/S at 37 °C without CO_2_.

### 4.3. LD Assay and Fatty Acid Uptake Assay

The accumulation of LDs was detected by BODIPY^®^ 493/503 dye (Thermo Fisher Scientific). LD accumulation was achieved by treating cells with OA conjugated to BSA. Cells were seeded in μClear^®^ 96-well plates (Greiner Bio-ONE, Frickenhausen, Germany) and loaded with OA with testing drugs or DMSO for the indicated periods. Cells were then fixed with paraformaldehyde and stained with 2 μg/mL Hoechst 33342 and 1 μg/mL BODIPY^®^ 493/503. Four (in screening) or nine (in further experiments) fields for each well were picked and images for nuclei and LD were acquired and analyzed automatically by an HCS instrument (ImageXpress Micro System, Molecular Devices, Sunnyvale, CA, USA). A granularity analyzing module was used to identify nuclei and LDs. The diameter settings for defining nuclei and LDs are 8–25 and 0.5–2 μm, respectively. For monitoring fatty acid uptake, BODIPY-labelled dodecanoic acid (BODIPY^®^ FL C12, Thermo Fisher Scientific) conjugated to BSA was used in the LD assay. For the LD accumulation kinetic study, Hoechst 33342 and BODIPY^®^ 493/503 were added at the same time as OA. Time-lapse images of living cells in each well were acquired once an hour for 24 h by an ImageXpress Micro System automatically.

### 4.4. RNA Isolation, Reverse Transcription (RT), and Real-Time PCR (qPCR)

Total RNA was isolated from cells using TRIzol (Life Technologies, Carlsbad, CA, USA) according to the manufacturer’s protocol. Complementary DNA (cDNA) was produced from cellular RNA (1 μg) using a high-capacity cDNA reverse transcription kit (Thermo Fisher Scientific). Real-time PCR reactions were performed using TOOLS Easy SYBR qPCR Mix (TOOLS, Taipei, Taiwan). Reactions were assayed using an Applied Biosystems StepOnePlus Real-Time PCR system. The primer pairs used are showed in Table 3.

### 4.5. Immunoblotting

Cells were lysed by RIPA lysis buffer and immunoblotting was performed as described previously [39]. The antibodies used were anti-CD36 (PA1-16813, Thermo Fisher Scientific) and anti-alpha Tubulin (66031-1-Ig, Proteintech Group, Chicago, IL, USA).

### 4.6. Statistical Analyses and Target Selection

All data were analyzed using GraphPad Prism 5.01 software (La Jolla, CA, USA). Differences between two groups were analyzed using Student’s *t*-tests. One-way analysis of variance (ANOVA) followed by Dunnett’s comparison test were used to compare differences in multiple groups. A *p*-value < 0.05 was considered statistically significant. The Z’-factor was used to quantify the suitability of the LD assay for the high-throughput screen [18]. A platform with a Z’-factor > 0.5 represents an excellent assay for HTS. The Z’-factor was calculated based on the following equation: Z’ = 1 − (3 SD of sample group + 3 SD of negative control)/(mean of sample group − mean of negative control). OA-treated wells were defined as general sample group and BSA-treated wells were defined as negative controls for calculating Z’-factor.

## Figures and Tables

**Table 1:** Statistical parameters of screening platform.

Parameters	Values
S/N ^a^	37.8
S/B ^a^	14.6
Z’-factor ^a^	0.66

^a^ Parameters were calculated as following: S/N = (mean of signal − mean of background)/SD (standard deviation) of background; S/B = mean of signal/mean of background; Z’ = 1 − (3 SD of sample group + 3 SD of negative control)/(mean of sample group − mean of negative control). Fatty acid free BSA-treated wells were considered as background, while OA-treated wells were defined as signal for S/N and S/N calculation. OA-treated wells were defined as general sample group and BSA-treated wells were defined as negative controls for calculating Z’-factor.

**Table 2:** IC_50_s and CC_50_s of SSLE in liver cell lines.

Cell Line	IC_50_ (μg/mL) ^a^	CC_50_ (μg/mL) ^a^
Huh7	30.7 ± 1.9	168.8 ± 6.6
HepG2	76.7 ± 1.6	>200
AML12	54.3 ± 1.7	81.9 ± 0.6
Clone9	7.6 ± 0.02	174.9 ± 3.6

^a^ IC_50_ and CC_50_ represent the half inhibitory concentration for LD inhibition and cell cytotoxicity of SSLE, respectively.

**Table 3:** Primers used in this study.

Gene	Species	Forward (5′>3′)	Reverse (5′>3′)
*CD36*	*Homo sapiens*	TCCTGCAGAATACCATTTGATCC	GGTTTCTACAAGCTCTGGTTCTTA
*CPT1*	*Homo sapiens*	TCCAGTTGGCTTATCGTGGTG	CTAACGAGGGGTCGATCTTGG
*SIRT1*	*Homo sapiens*	GCGGTTCCTACTGCGCGA	TCACTAGAGCTTGCATGTGAGG
*DGAT1*	*Homo sapiens*	CAACAAGGACGGAGACGCCGG	GATGCCACGGTAGTTGCTGAAGCC
*APOB*	*Homo sapiens*	ACCTCCAGAACATGGGATTGC	GGGCTGGTGTCCTAACAGTC
*MTTP*	*Homo sapiens*	TGAGGCAGTGGCCATAGAAAAT	CTTTGTCTTGATGAGCCTGGTA
*PGC1a*	*Homo sapiens*	GTCACCACCCAAATCCTTAT	ATCTACTGCCTGGAGACCTT
*TBP*	*Homo sapiens*	CAGAAGTTGGGTTTTCCAGCTAA	ACATCACAGCTCCCCACCAT
*Cd36*	*Mus musculus*	CCTTAAAGGAATCCCCGTGT	TGCATTTGCCAATGTCTAGC
*Gapdh*	*Mus musculus*	CTGCACCACCAACTGCTTAGC	GGTCATGAGCCCTTCCACAAT

## Supplementary Materials

Supplementary materials can be found at http://www.mdpi.com/1422-0067/19/7/2130/s1.

## Abbreviations

NAFLD	non-alcoholic fatty liver disease
LD	lipid droplet
HTS	high-throughput screening
NASH	nonalcoholic steatohepatitis
T2DM	Type 2 diabetes mellitus
CVD	cardiovascular disease
CKD	chronic kidney disease
HCC	hepatocellular carcinoma
TG	triglyceride
TIP	Taiwanese Indigenous Plant
HCS	high content screening
FBS	Fetal Bovine Serum
OA	oleic acid
BSA	bovine serum albumin
RT	reverse transcription
qPCR	real-time PCR
S/N	signal-to-noise ratio
S/B	signal-to-background ratio
TC	triacsin C
SD	standard deviation
ACSL	long-chain fatty acyl-CoA synthetase
VLDL	very-low-density lipoprotein
DGAT1	diacylglycerol *O*-acyltransferase 1
CPT1	carnitine palmitoyltransferase I
APOB	apolipoprotein B
MTTP	microsomal triglyceride transfer protein
SIRT1	sirtuin 1
PGC1a	peroxisome proliferator-activated receptor gamma coactivator 1-alpha
NAFLD	non-alcoholic fatty liver disease
LD	lipid droplet
