# Supplementary material for: High-Content Screening of a Taiwanese Indigenous Plant Extract Library Identifies Syzygium simile leaf Extract as an Inhibitor of Fatty Acid Uptake
Source: Int J Mol Sci. 2018 Jul 22;19(7):2130. doi: 10.3390/ijms19072130 (PMC6073993; doi:10.3390/ijms19072130)
Supplement: Supplementary file 1 [file ijms-19-02130-s001.zip › Supplementary.pdf]

Supplementary Materials: High-Content Screening of a Taiwanese Indigenous Plant Extract Library Identifies *Syzygium simile* leaf Extract as an Inhibitor of Fatty Acid Uptake

Chia-Hung Yen, Hsun-Shuo Chang, Tsai-Hsun Yang, Sheng-Fan Wang, Ho-Cheng Wu, Yu-Chang Chen, Kai-Jay Lin, Sheena Wang

Table S1. Number of taxa of Taiwan indigenous plant extract library by major plant group.

Classification	*N* (%)
Ferns	140 (10.5)
Gymnosperm	12 (0.9)
Dicotyledons	1,103 (82.5)
Monocotyledons	81 (6.1)

Table S2. Skeleton classification of compounds isolated from *Syzygium* genus.

Skeleton	Number	Skeleton	Number
Flavonoids	58	Aromatic hydroxyketones	4
Galloyl glucoses	24	Phenylpropanoids	4
Triterpenoids	24	Megastigmanes	3
Phloroglucinol derivatives	11	Alkaloids	3
Chalcones	10	Chromones	3
Steroids	10	Alkanes	2
Lignans	9	Monoterpenoids	2
Benzenoids	8	Neolignan	1
Gallic acids	7	Isoflavonoid	1
Phenol glucoside gallates	7	Diterpenoid	1

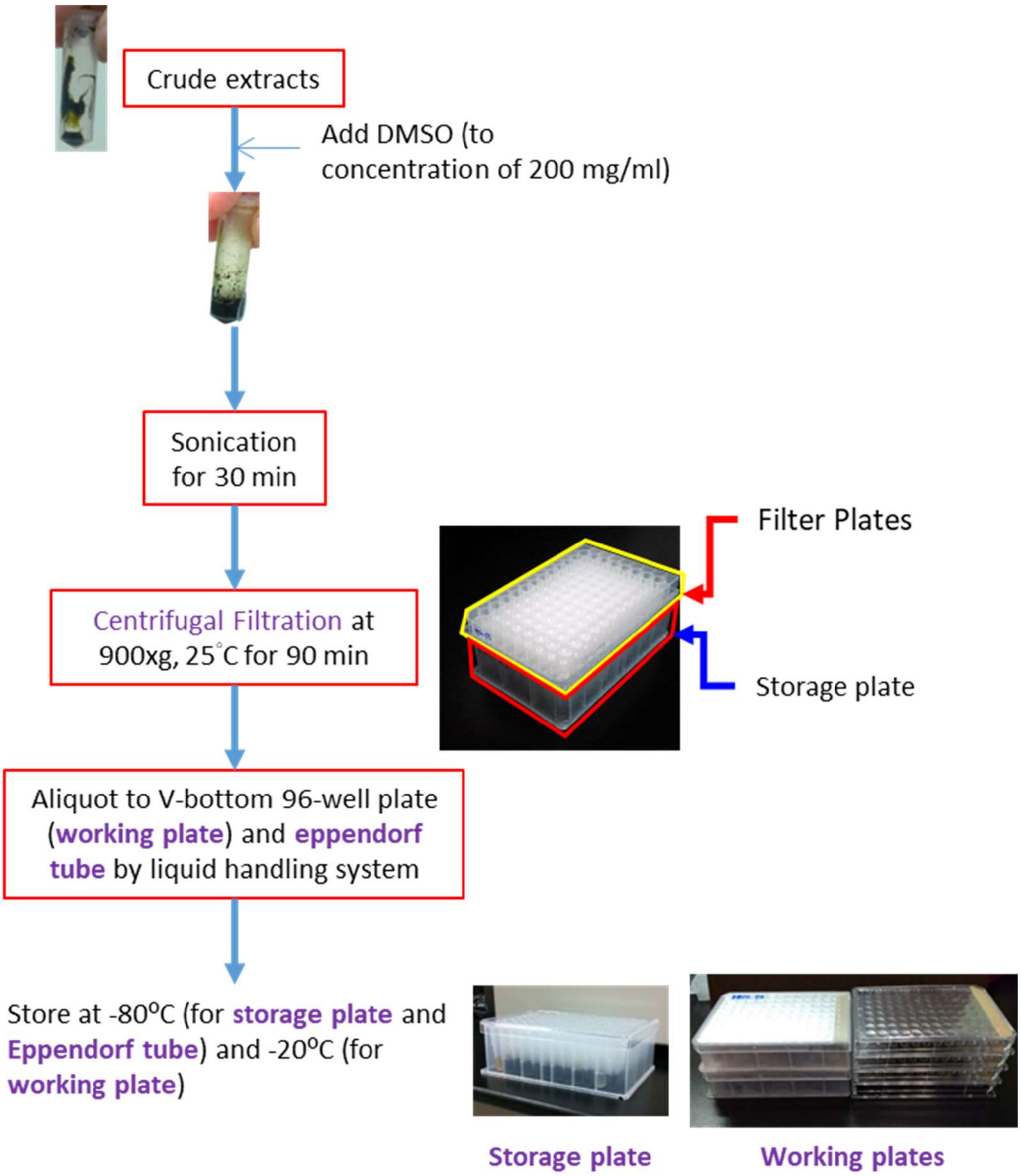

Figure S1. Flowchart of library construction. The dried crude extracts were dissolved in DMSO at the concentration of 200 mg/mL, sonicated for 30 min at 25 °C, and then the unsolvable residuals were removed by centrifuge filtration with the MultiScreen® Solvinert Filter Plate (0.45 μm PTFE membrane). The flow-through (dissolved extract) were collected in 96-Well MASTERBLOCK® plate. The extracts were then transferred into V-bottom 96-well plate and Eppendorf tube by a liquid handling system. The storage and working plates were sealed with foil and stored at −80 and −20 °C, respectively. After screening hit extracts in the Eppendorf tube aliquot will be used in further validating experiments.

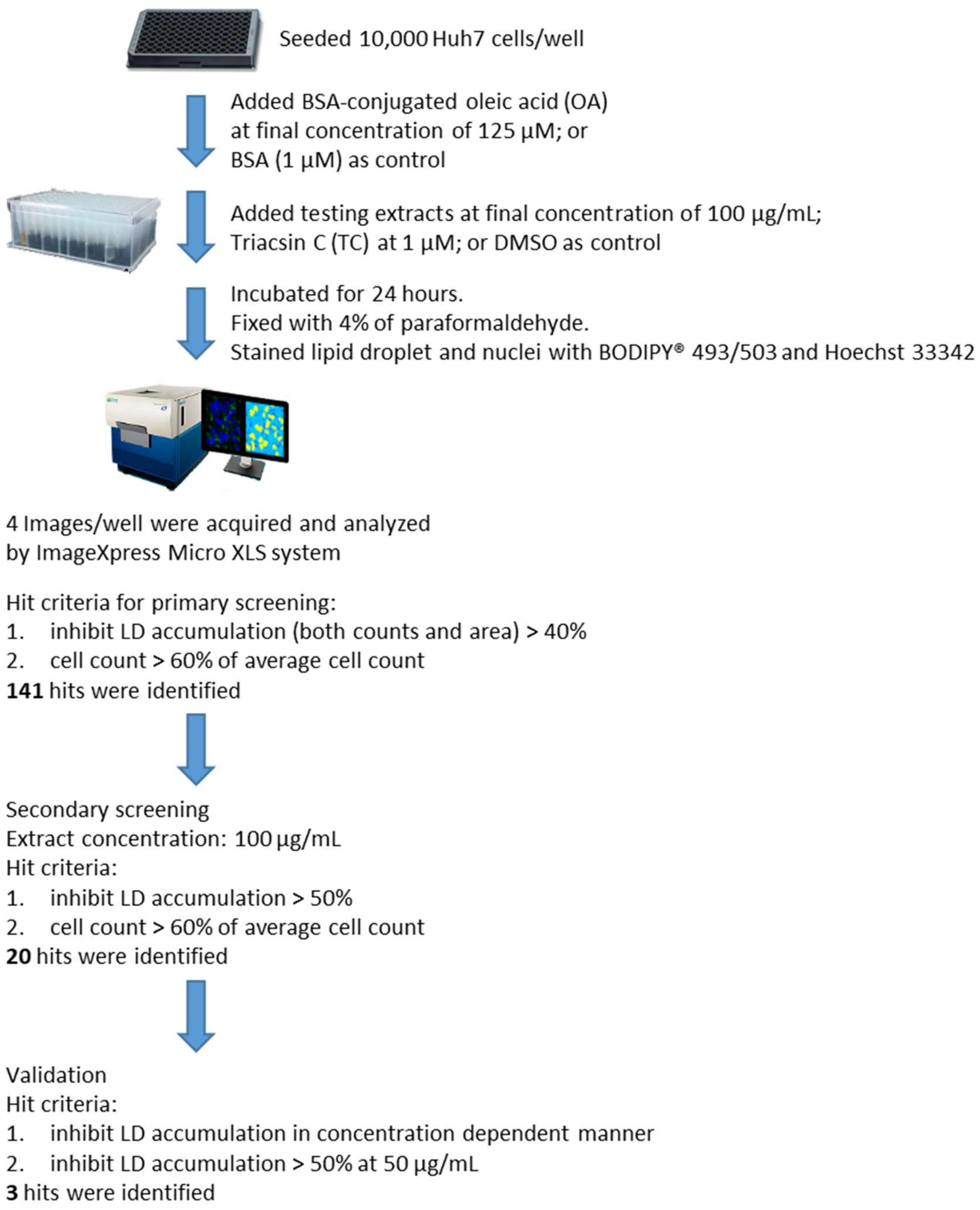

Figure S2. Flowchart of screening.

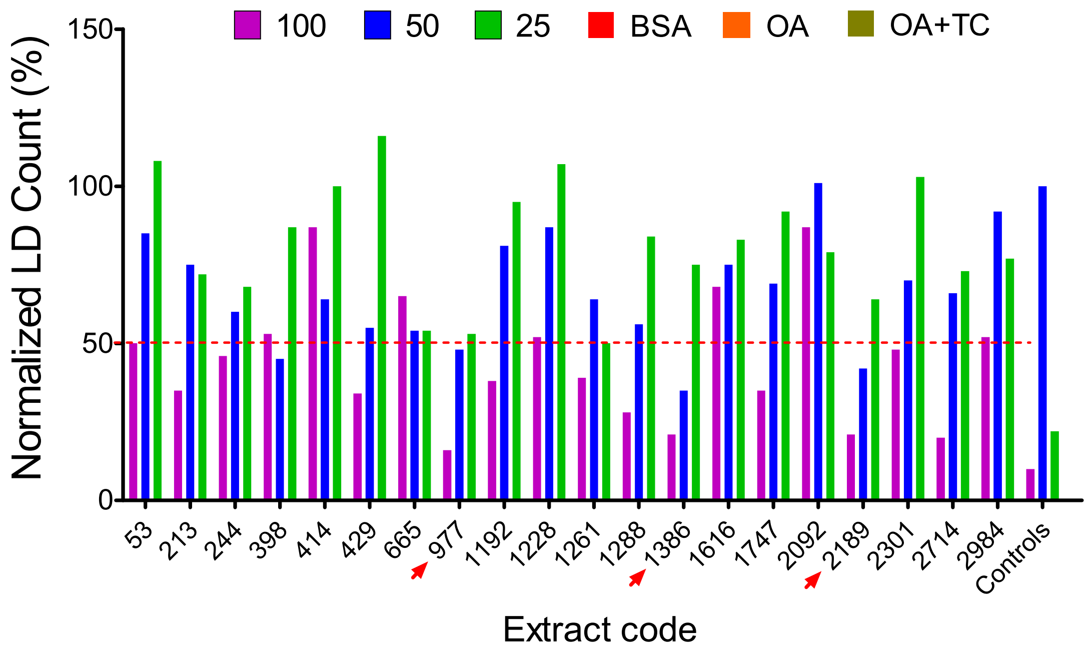

Figure S3. Results of Validation experiment. Huh7 cells were treated with BSA or 125 μM OA in the absence or presence of SSLE at 25, 50, and 100 μg/mL or 1 μM TC. After 24 h, LD counts were determined as described in Materials and Methods section. Extracts that reduced LD content in concentration-dependent manner and more than 50% (below red dash line) at 50 μg/mL were considered as the final hits for HTS (indicated by red arrow). Extract code no.977 is the methanolic extract prepared from the leaves of *Syzygium simile* (SSLE).

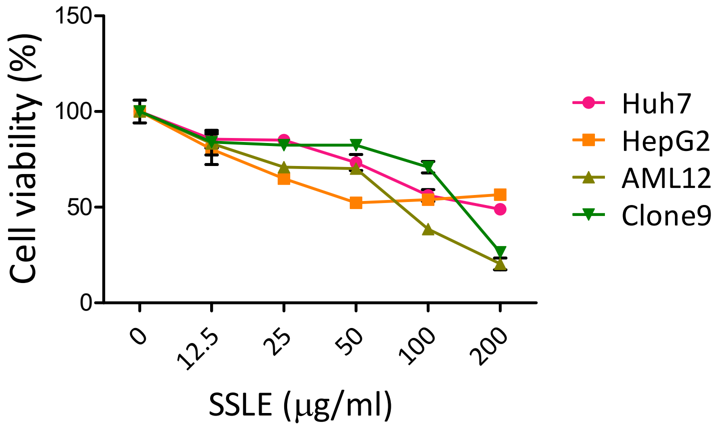

Figure S4. The cytotoxicity of SSLE in 4 hepatic cell lines. Liver cell lines from human (Huh7 and HepG2), mouse (AML12) and rat (Clone9) were used to test the cytotoxicity of SSLE. Cells were treated with a series concentration of SSLE for 72 h. Cell viability was measured by alamarBlue® reagent according to the manufacturer's protocol. The results were used to calculate half concentration for cell cytotoxicity (CC_{50}) of SSLE by using GraphPad Prism 5.01 software.

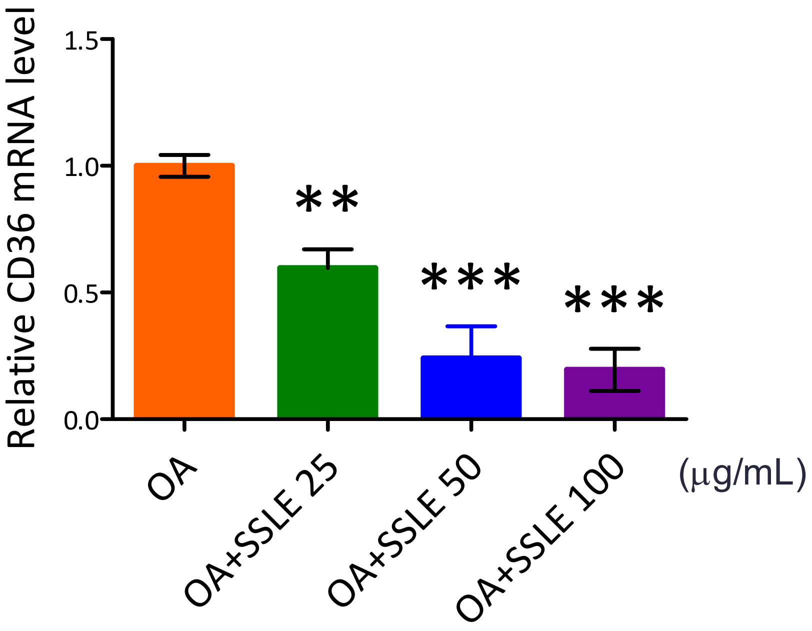

Figure S5. Dose-dependent repression of CD36 mRNA expression by SSLE. Huh7 cells were treated with 125 μM OA in the presence or absence of SSLE at indicated concentrations for 12 h. RT-qPCR was used to determine the expression CD36 gene.

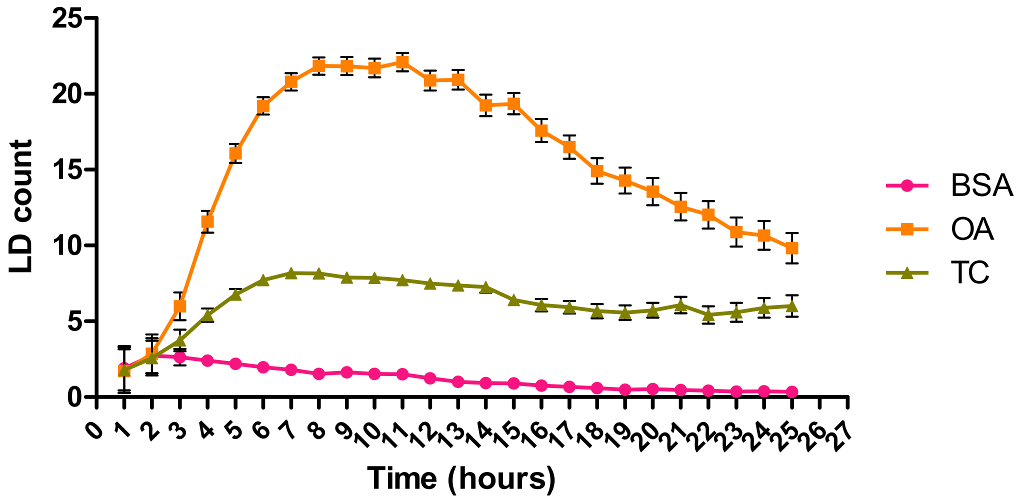

Figure S6. Kinetics of LD formation. Huh7 cells seeded in 96-well plate were treated as indicated in the presence of staining reagents. Then the time-lapse images of living cells were acquired once an hour for 25 h by ImageXpress Micro System automatically. The original videos were shown in Video S1–S3.

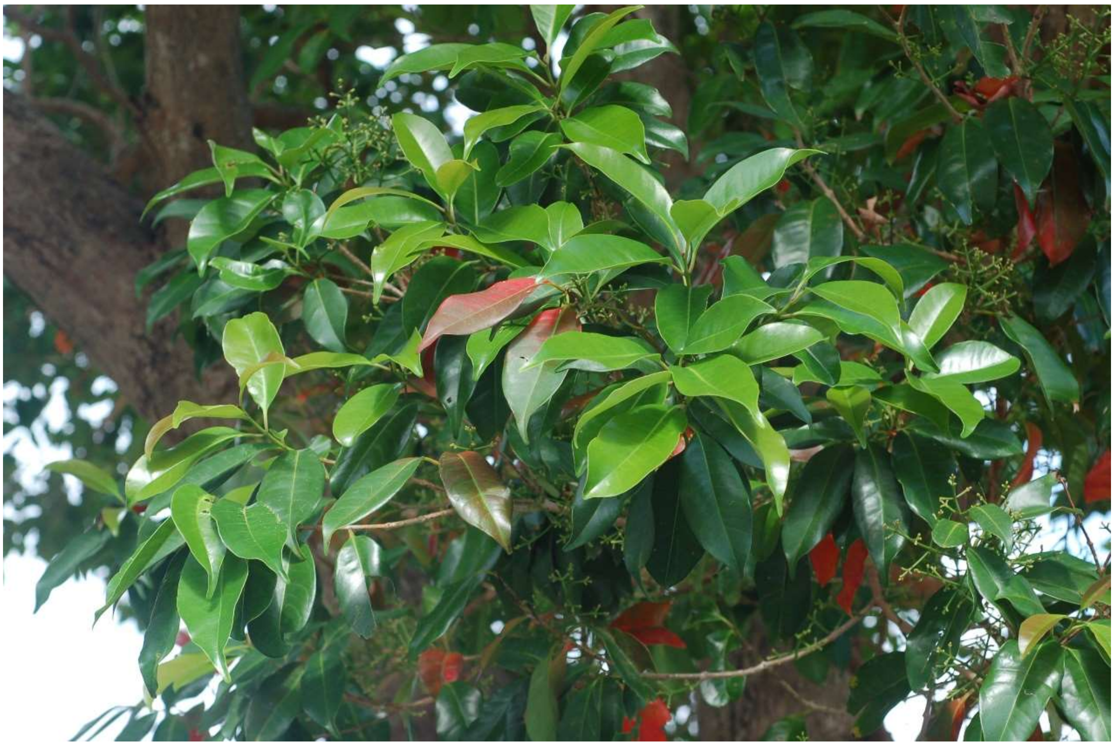

Figure S7. Picture of *Syzygium simile*.
